# Copper–Collagen Interactions Regulate the Mechanical and Invasive Properties of Tumor Spheroids

**DOI:** 10.1002/adhm.202505120

**Published:** 2026-04-09

**Authors:** Paula Guerrero‐López, Jose I. Garcia‐Peiro, Felipe Hornos, Jose L. Hueso, Jesus Santamaria, J. Manuel Garcia‐Aznar

**Affiliations:** ^1^ Multiscale in Mechanical and Biological Engineering (M2BE) Aragon Institute of Engineering Research (I3A) University of Zaragoza Zaragoza Spain; ^2^ Instituto de Nanociencia y Materiales de Aragon (INMA) Campus Rio Ebro CSIC‐Universidad de Zaragoza Zaragoza Spain; ^3^ Department of Chemical and Environmental Engineering Campus Rio Ebro University of Zaragoza Zaragoza Spain; ^4^ Networking Research Center in Biomaterials Bioengineering and Nanomedicine (CIBER‐BBN) Instituto De Salud Carlos III Madrid Spain; ^5^ Instituto de Investigación Sanitaria (IIS) de Aragón Zaragoza Spain; ^6^ Instituto de Investigación Desarrollo e Innovación en Biotecnología Sanitaria de Elche (IDiBE) Universidad Miguel Hernández 03202 Elche Spain; ^7^ Escuela Politécnica Superior Universidad de Zaragoza Huesca Spain

**Keywords:** 3D cultures, cancer, collagen, copper, microchips

## Abstract

Copper is a key trace element with established cytotoxic properties, yet its interactions with the extracellular matrix and their implications for the evolution of 3D culture models remain poorly understood. Here, we study how copper entrapped in collagen can regulate the structural and invasive properties of 3D tumor spheroids. Our findings reveal that copper influences cytoskeletal organization, protrusion dynamics, and epithelial–mesenchymal transitions. Interestingly, we also highlight its dual capacity to either suppress or enhance invasive behavior depending on the context of exposure. These results position copper as a context‐dependent modulator of tumor progression and underscore the importance of dose and timing in determining therapeutic outcomes. By integrating a physiologically relevant 3D model, this study provides new insights into copper–ECM interactions and identifies potential strategies to exploit them for cancer therapy.

## Graphical abstract

Ionic copper is investigated as a modulator of tumor spheroid growth and invasiveness in a 3D microfluidic glioblastoma model. Copper exposure alters metabolic activity, cytoskeletal organization, protrusion dynamics, and EMT‐related phenotypes in a dose‐ and timing‐dependent manner, revealing how copper bioavailability shapes tumor aggressiveness within a confined extracellular matrix.

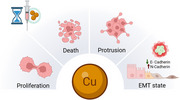

## 1 Introduction

Copper is an essential trace element that plays a critical role in various biological functions, yet it exhibits a dual nature in living systems [1, 2, 3]. While small amounts are indispensable for enzymatic activity and cellular processes, excess copper is highly toxic [4, 5]. This toxicity arises through multiple mechanisms, many of which remain under active investigation [6]. One well‐established involves disruption of redox homeostasis. In this case, copper promotes the generation of reactive oxygen species (ROS) via hydrogen peroxide activation and glutathione (GSH) oxidation, leading to oxidative damage to cellular components [7, 8, 9]. Additionally, copper toxicity has been linked to regulated cell death pathways, such as ferroptosis, a metal‐dependent lipid peroxidation‐induced cell death [10], and the more recently identified cuproptosis, where mitochondrial copper accumulation triggers protein aggregation and cellular dysfunction [11].

These distinct mechanisms illustrate copper´s complex role in modulating cell viability, particularly under pathological conditions like cancer, where dysregulated copper homeostasis can drive tumor progression or induce cell death [11, 12]. However, the precise conditions under which copper evolves from a vital cofactor to a toxic agent remain poorly defined [13, 14]. Notably, increasing copper levels in the tumor microenvironment (TME) has been proposed as a potential therapeutic strategy exploiting copper's ability to disrupt cancer cell homeostasis [15, 16, 17, 18, 19]. Despite growing understanding of copper´s intracellular effects, its mechanochemical and mechanobiological roles in tumors remain underexplored. Few studies have examined how extracellular copper influences cancer progression or how interactions between copper and the extracellular matrix (ECM) modulate tumor behavior [20].

This work attempts to contribute to this area. Tumor mechanical properties, including their ability to invade adjacent tissues and remodel the TME, are increasingly recognized as therapeutic targets, as their modulation can limit disease progression and enhance treatment efficacy [21, 22]. Mechanotherapy emerges as a strategy to exploit the physical characteristics of tumors to interfere with their growth [22]. In this context, investigating copper–ECM interactions, particularly with structural proteins like collagen type I, may provide insight into new mechanisms regulating cancer cell migration, invasion, and proliferation [20, 23, 24].

To study these effects, experimental models that allow in situ monitoring of tumor growth while retaining enough key features to reproduce their behavior are essential. 3D spheroid cultures embedded in collagen‐based ECM represent a promising platform [25, 26]. These models balance simplicity and behavior emulation, enabling physiologically relevant investigation of cell–cell and cell–matrix interactions, as well as the mechanical dynamics of tumor progression over time [25, 26]. Using such 3D spheroids, in this work we systematically examine the impact of extracellular copper on tumor growth, invasiveness, and migration, with the aim of elucidating the biomechanical consequences of copper–ECM interactions in the TME.

## 2 Materials and Methods

### 2.1 Materials

Copper (II) chloride dihydrate (CuCl_2_·2H_2_O, ≥ 99.0%), bovine serum albumin (BSA), phosphate‐buffered saline (PBS) buffer (pH 7,4), poly‐D‐lysine (PDL), and phalloidine were purchased from Sigma–Aldrich. Water was obtained from a Milli‐Q Advantage A10 System with resistivity of 18.2 mΩ (Merck Millipore, Germany). Polydimethylsiloxane (PDMS, Sylgard 184) was obtained from Dow Corning GmbH, Dulbecco's modified Eagle's medium (DMEM) and FBS from Gibco, and collagen type I (rat tail high concentration) from Corning. Antibodies for N‐Cadherin (Mouse mAb) and E‐Cadherin (Rabbit mAb) were purchased from Cell Signaling Technology, and their respective secondary antibodies, Alexa Fluor 647 (Goat anti‐mouse IgG) and Alexa Fluor 488 (Goat anti‐Rabbit IgG), as well as SYTOX Green Nucleic Acid Stain from Life Technologies (ThermoFisher). Invitrogen supplied Dapi.

### 2.2 Characterization Techniques

Elemental analysis was carried out with an EDX detector for energy‐dispersive spectroscopy experiments in scanning mode. EDX mappings were acquired with an Oxford Instruments (NanoAnalysis & Asylum Research, High Wycombe, UK) detector and analyzed with the built‐in AZtec software. X‐Ray photoelectron spectroscopy (XPS) was performed with an Axis Supra spectrometer (Kratos Tech). The samples were mounted on a sample rod placed in the pretreatment chamber of the spectrometer and then evacuated at room‐temperature. The spectra were excited by a monochromatized Al Kα source at 1486.6 eV and subsequently run at 8 kV and 15 mA. A survey spectrum was measured at 160 eV of pass energy, and for the individual peak regions, spectra were recorded with a pass energy of 20 eV. Analysis of the peaks was performed with the CasaXPS software using a weighted sum of Lorentzian and Gaussian component curves after Shirley background subtraction. The binding energies were referenced to the internal C 1s standard at 284.5 eV.

### 2.3 Copper Release Kinetics

Special attention was focused on the capability of the collagen‐based hydrogel used in this work to sequester Cu ions. To study this aspect, a collagen‐based hydrogel was synthesized following the protocol developed by Shin et al. [27]. Briefly, 400 µL of collagen solution (6 mg mL^−1^) in DMEM was incubated at 37°C for 20 min for hydrogel polymerization in 3 mL wells. Then, 1 mL of copper chloride solution (0.05 mg mL^−1^) was added to the hydrogel. 600 µL of supernatant solution was collected for further elemental analysis. At the experiment endpoint, the hydrogels were analyzed together to close mass balances and elucidate how much metal moved to the solution. All the samples were digested with HCl:HNO_3_ (3:1) mixture overnight. Cu concentrations were determined through elemental analysis with Agilent 4100 MP‐AES.

### 2.4 Microfluidic Device Fabrication

The microfluidic devices used are made of PDMS with a design consisting of a central chamber where one hydrogel, which recreates tissue matrix, is confined and two side channels through which the nutrients are introduced. The geometry was adjusted in an SU‐8 master mold on a silicon wafer, from which it was replicated with PDMS. This material is fabricated with a 10:1 weight ratio mixture of base and curing agent, cured in an oven at 80°C, and then cropped and punched to make the access to the channels. Finally, the PDMS devices were attached to the glass bottom of 35 mm Petri dishes by activating the surfaces with a plasma treatment, and later, they were treated with PDL to improve the adhesion of the collagen matrix to the device.

### 2.5 Hydrogel Preparation and Cell Seeding

The human GBM cell line U251‐MG was cultured with DMEM at 4.5 g L^−1^ glucose and supplemented with 10% FBS. Cells were incubated at 37°C with 5% CO_2_ until 80% confluence was reached for use in the experiment. For seeding in the 3D culture, they were trypsinized, centrifuged (1200 rpm, 5 min), and passed through a 40 µm cell strainer to ensure the removal of cell aggregates. Subsequently, cells were counted using a Neubauer chamber and added to the collagen mix to leave a final concentration of 0.2 × 10^6^ cell mL^−1^. The 3D cell culture was developed in a type I collagen‐based matrix using the protocol by Shin et al. [27]. Following these indications, the hydrogel consisted of a mixture at 4°C of 10X DPBS, collagen at a final concentration of 6 mg mL^−1^, and 0.5 m NaOH to adjust pH to 7.5. This mix was introduced into the central chamber and left to polymerize at 37°C in humid boxes, turning the device every 5 min for at least 20 min. Finally, the lateral channels were hydrated with a culture medium periodically. When required, copper treatments at 0.05, 0.025, 0.01, or 0.005 mg mL^−1^ were administered by dissolving directly into the culture medium, either from the beginning of the experiment or starting at day 6.

### 2.6 Image Acquisition and Analysis

The spheroid growth was monitored with a Leica DM IL Led microscope. Photos were taken of the central chamber daily at 4X magnification in brightfield. Later, these images were processed, and the spheroid area was segmented with the semiautomatic Segmentation3D App developed by C. Borau using MATLAB (Mathworks, Natick, CA, US) as described by Alamán‐Díez et al. [28]. The data obtained were processed and represented using GraphPad Prism 8. For the protrusion analysis, 12 h time lapses after 9 days of treatment were performed with a Carl Zeiss Axio Observer Z1 7. Photos were acquired every 20 min at 40X magnification in brightfield at 37°C with 5% CO_2_, and the measurements of protrusion length were performed with ImageJ. 2D structure fluorescence images and HIF‐1α expression images were obtained using the Nikon D‐Eclipse C1 confocal microscope equipped with a Plan Apo VC 40XH objective, and for 3D reconstruction of the cytoskeleton and Cadherins expression, a ZEISS Lattice Lightsheet 7 microscope was used at 40X magnification. Fluorescence image analysis was performed with ImageJ.

To assess potential alterations in collagen fibre organization caused by copper treatment, second‐harmonic generation (SHG) multiphoton microscopy (Stellaris‐Dive; Leica) was employed at 20X magnification at the end‐point. This technique allows the label‐free visualization of collagen fibres by exploiting their nonlinear optical properties. For each condition, three representative ROIs were imaged. Spheroids were co‐imaged with vimentin to provide a cellular context.

### 2.7 Immunofluorescence Staining

For the structure analysis, the samples were stained with DAPI and Phalloidine. To begin with, samples were fixed with 4% paraformaldehyde in PBS for 15 min, and to remove it, 5 min washes were performed three times. To permeabilize cell membranes, samples were treated for 10 min at room‐temperature with 0.1% Triton X‐100 in PBS and, after that, washed three times with PBS for 5 min. Blocking was done with 5% bovine serum albumin in PBS overnight at 4°C. Later, Phalloidine and DAPI were added to samples, both of them diluted 1:100 in PBS, and incubated for 4 h at room‐temperature in darkness. Cells were washed three times for 5 min with PBS again. For the EMT study, E‐ and N‐Cadherin were stained in spheroids, using Alexa 488 and 647, respectively. The staining protocol is similar to structure one, with primary and secondary antibodies. Fluorescence intensity was measured with Matlab. Vimentin staining was performed following the same protocol, using a primary antibody diluted 1:300 and an Alexa Fluor 488–conjugated secondary antibody.

### 2.8 Viability and Stress Measurements

In order to measure viability, Alamar Blue reagent (ThermoFisher Scientific) was used mixed with normal DMEM medium (1:9) at the end‐point. After 4 h of incubation at 37°C, the mixture was collected from the microdevices and its fluorescence was measured using a plate reader with a fluorescence excitation wavelength of 540–570 nm and emission at 580–610 nm. To establish the relationship between Alamar Blue fluorescence and cell number, a calibration curve was constructed across a range from 20 000 to 0 cells.

For the metabolic stress assessment, HIF‐1α expression was measured using Image‐iT Red Hypoxia reagent (ThermoFisher Scientific). The reagent was added through the lateral channels diluted in DMEM media at a final concentration of 10 µm and incubated for 1 h at 37°C. Finally, the media was removed and fresh media was added.

### 2.9 Statistical Analysis

Each condition underwent triplicate testing. Statistical analysis was conducted using GraphPad Prism 8 and expressed as the mean ± SEM. The normality of the data was assessed using the Shapiro–Wilk test. Analysis of variance (ANOVA) was then performed, followed by post hoc Dunnett tests to ascertain statistical significance across the continuous variables under different conditions. Two‐way ANOVA followed by Dunnett multiple comparison test was used to analyze data with more than one variable. In cases where data distribution was non‐normal, nonparametric Kruskal–Wallis tests were employed, followed by post hoc Dunn's tests. All statistical tests performed are two‐tailed, and a p‐value of < 0.05 is considered significant.

## 3 Results

### 3.1 Characterization of Collagen–Copper Ion Interactions

**FIGURE 1:**
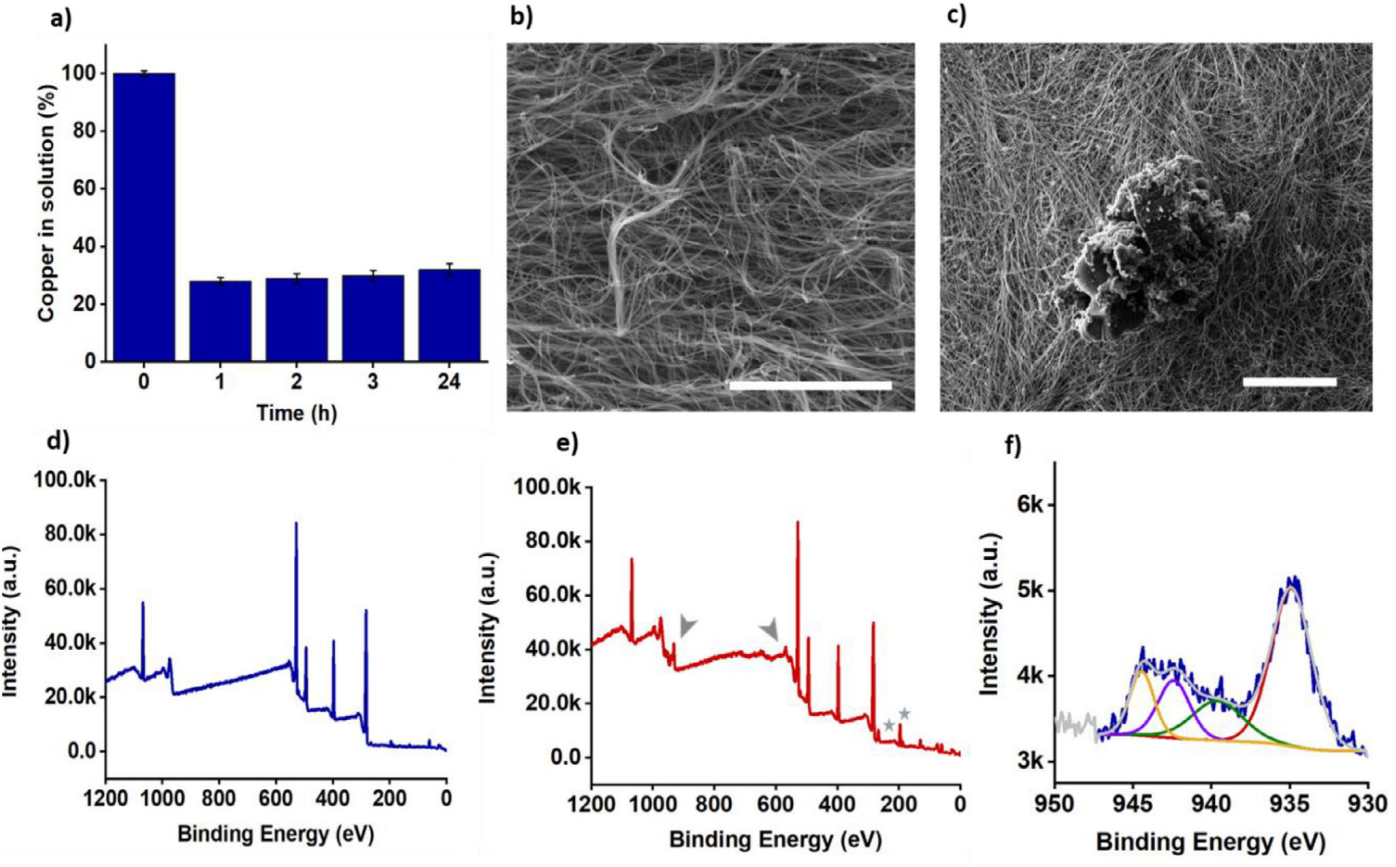
Collagen characterization in the presence of copper: (a) relative copper concentration in solution when exposed to collagen over time. Cryo‐SEM images of collagen‐based matrices in the absence (b) and presence (c) of spheroids (Scale bar is 10 µm). wide‐scan XPS spectrum of dried, untreated collagen with characteristic C 1s, N 1s, and O 1s peaks among others, and (e) treated collagen, where extra peaks associated with copper are observed. (f) High‐resolution scans of the Cu 2p region.

To evaluate the interaction between copper ions and collagen, we first quantified elemental copper remaining in collagen samples treated with CuCl_2_. Polymerized collagen hydrogels (2.4 mg) were incubated with copper ions (0.05 mg of total copper dissolved), and copper content in the supernatant was measured as a function of time. As shown in Figure 1a, copper concentration in the solution sharply dropped to 25% after 1 h, indicating rapid adsorption of copper ions by the matrix. After 24 h, the remaining copper in solution approached a stable value of 32%, suggesting strong and sustained collagen–copper interactions. To confirm this, we performed a complementary experiment using an analogous collagen/copper ratio but replacing the supernatant at fixed time intervals, after the initial uptake of Cu by the collagen matrix. As shown in Figure S1, only minimal copper release was observed, and this decreased with successive media changes. During the first cycle, approximately 5% of copper was released from the collagen after 8 h. In the subsequent two cycles, the amount of copper released decreased to 2% and 1%, respectively. These results suggest that most of the Cu in the collagen (> 90%) is strongly bound and is retained even after prolonged contact with a Cu‐free medium. Only in the first release cycle, a relatively important amount of Cu is released, corresponding to either weakly bound or interstitial Cu. Once this easily available Cu is exhausted, successive cycles extracted much less Cu, highlighting the capacity of the collagen matrix to retain copper ions. We hypothesized that this feature could offer an interesting scenario to study the impact of Cu on spheroid development. The density and stiffness of the extracellular matrix (ECM) have been reported to have a strong effect on spheroid formation and invasion [29, 30]. In this case, we include Cu, with a strong interaction with the collagen that is a main component in the ECM, and monitor its influence on spheroid evolution.

We investigated the morphological properties of the collagen‐based hydrogels using reflection microscopy (Figure S2) and cryo‐scanning electron microscopy (cryo‐SEM) of spheroids cultured in collagen matrices (Figure 1b,c). Interestingly, energy‐dispersive X‐Ray spectroscopy (SEM‐EDX) did not detect significant levels of copper. Since typical detection limits for Cu are below 1 wt.%, this suggests a homogeneous distribution of copper within collagen fibers (Figure S3).

To further assess copper incorporation, X‐ray photoelectron spectroscopy (XPS) was conducted on both untreated and copper‐treated matrices. As shown in Figure 1d, the wide‐scan XPS spectrum of dried, untreated collagen revealed characteristic C 1s, N 1s, and O 1s peaks, consistent with its organic composition. In contrast, the XP spectrum of collagen treated with CuCl_2_ (Figure 1e) displayed additional binding energy peaks centered at ∼932 and ∼952 eV, corresponding to Cu 2p_3_/_2_ and Cu 2p_1_/_2_, confirming copper incorporation [31]. Features at ∼570 eV were attributed to Cu LMM Auger transitions [32]. Chloride peaks at ∼200 eV (Cl 2p) and ∼266 eV (Cl 2s) confirmed the presence of residual precursor salts. High‐resolution scans of the Cu 2p region (Figure 1f) revealed a peak at 934.9 eV, accompanied by intense satellite signals from 940–945 eV, consistent with Cu^2^^+^ complexes (Figure 1f) [33]. Having established the interaction between copper and collagen, we next evaluated how this interface affects spheroid growth and proliferation.

### 3.2 Impact of Copper on Spheroid Growth Dynamics

**FIGURE 2:**
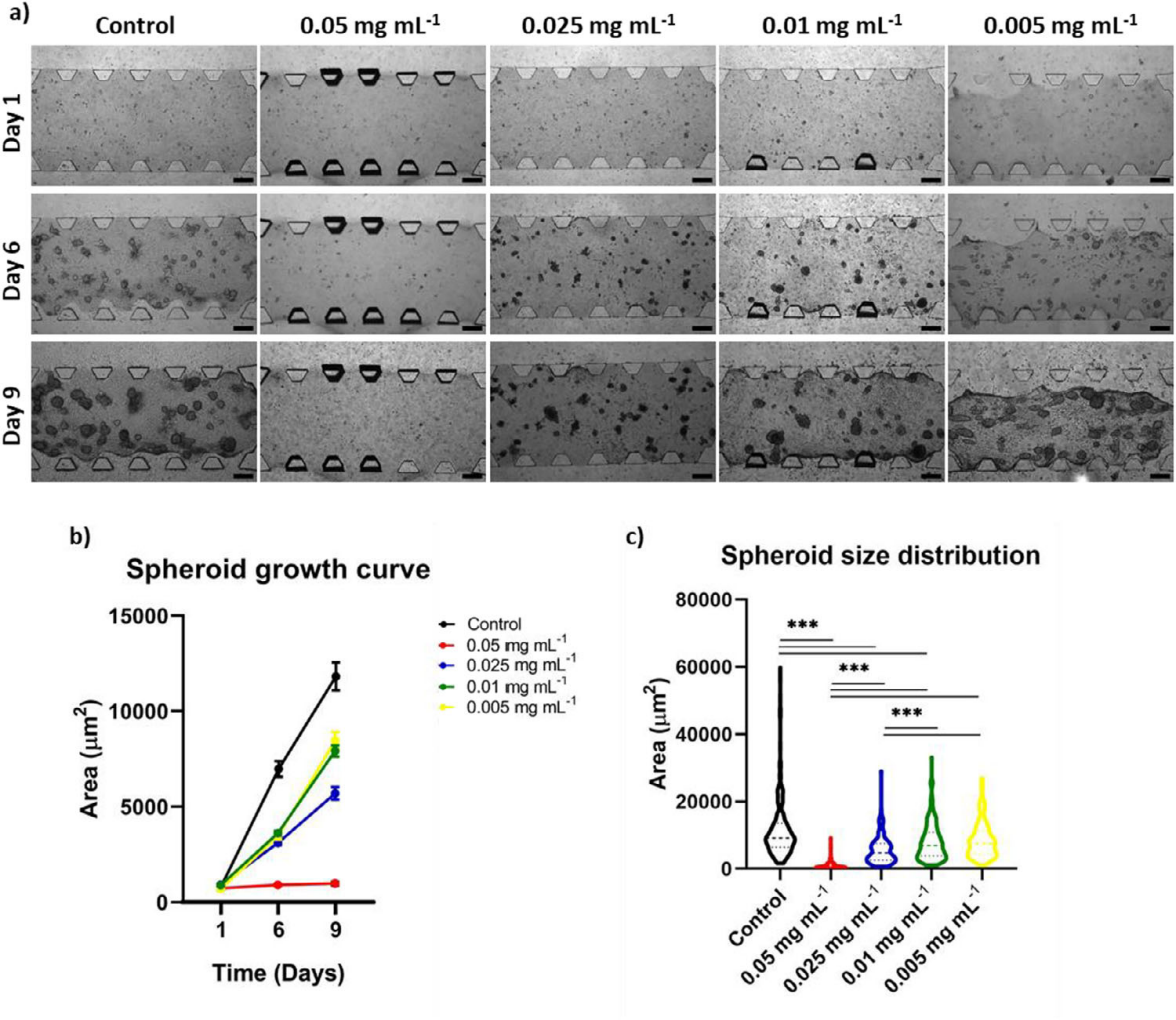
Influence of ionic copper treatment in 3D tumor growth: (a) Brightfield microscopy along 9 days (*n* = 2) allows the comparison of the growth of the 3D cellular cultures when exposed to a total concentration of 0.05, 0.025, 0.01, and 0.005 mg mL^−1^ of ionic copper. Scale bar is 250 µm. Images were processed and segmented to obtain the growth curve (b) and population size distribution at endpoint (c). Data shown as the mean ± sem (*n* = 3 technical replicates, and *n* = 2 experimental replicates); ^*^ *p*‐val < 0.033; ^**^ *p*‐val < 0.002; ^***^ *p*‐val < 0.001.

Increasing concentrations of ionic cooper were tested in a 3D cell culture model to assess their effects on tumor spheroid development, serving as a simplified representation of tumor progression. U251‐MG glioblastoma cells were embedded within the collagen hydrogel, and copper ions were delivered through the lateral channels, allowing cells to proliferate and self‐organize into 3D spheroids within the matrix architecture. Figure 2a shows brightfield microscopy images of the central chamber of the microfluidic devices at different times. The chips were filled with the collagen matrix, and the U251‐MG cells were seeded and allowed to form spheroids, whose growth was monitored in the central chamber over time to evaluate the effects of copper treatment on glioblastoma proliferation. Under ionic copper exposure, spheroids of varying sizes developed, with statistically significant differences observed between treated and control groups (Figure 2a). Periodic brightfield imaging (Figure 2a) revealed an inverse relationship between copper concentration and spheroid growth. (Figure 2a). In particular, the highest concentration (0.05 mg mL^−1^) led to a notable loss of cell viability, as evidenced by the absence of spheroid expansion and signs of cellular disaggregation.

This observation was quantitatively supported by the spheroid growth curve over time, obtained through segmentation of the brightfield images (Figure 2b). In control experiments, spheroids exhibited sustained growth over time, as observed in the left column of Figure 2a and in the corresponding curve of Figure 2b. A comparable but attenuated growth was observed at 0.005 mg mL^−1^, followed closely by the 0.01 mg mL^−1^ condition. In contrast, spheroids treated with 0.025 mg mL^−1^ grew similarly to controls until day 6, after which growth was arrested, resulting in significantly smaller final sizes by day 9. Notably, the 0.05 mg mL^−1^ treatment resulted in a flat growth curve, consistent with negligible proliferation and extensive cell death.

At day 9, spheroid size distributions (Figure 2c) differed significantly between controls and all copper‐treated groups, except for 0.005 mg mL^−1^, which largely overlapped with control sizes. As expected, 0.025 and 0.05 mg mL^−1^ treatments significantly reduced spheroid size compared to both control and lower‐dose groups (0.005 and 0.01 mg mL^−1^), which showed no significant difference from each other. Additionally, spheroids viability and stress‐related markers were evaluated at the end‐point (Figure S4). Alamar Blue measurements revealed significantly higher metabolic activity in control spheroids compared to copper‐treated conditions.

### 3.3 Assessment of Invasive Properties of Spheroids Under Copper Influence

**FIGURE 3:**
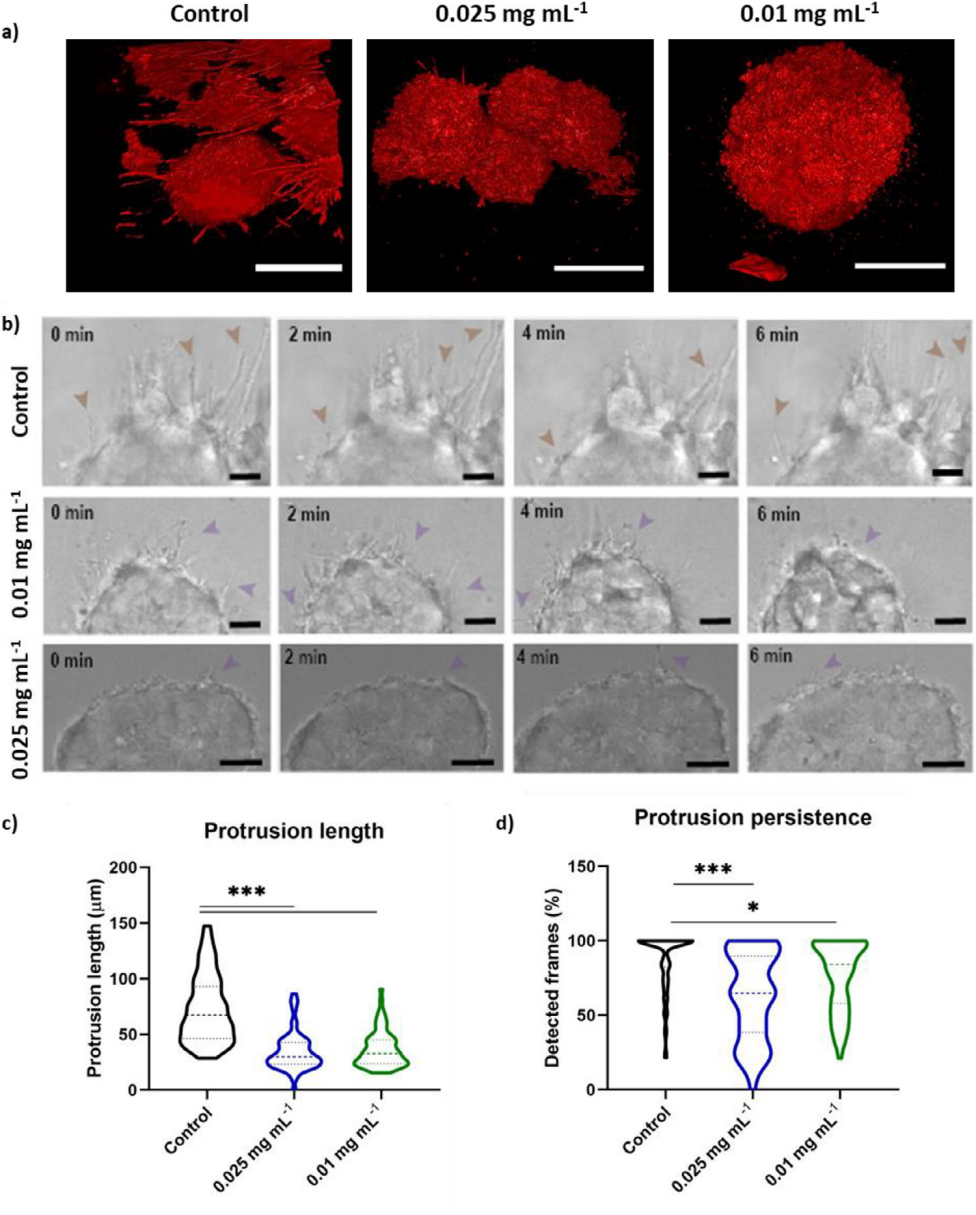
Influence of ionic copper treatment in protrusion formation and development in 3D tumors: (a) 3D tumor reconstruction of confocal images of phalloidin‐stained GBM spheroids treated with different ionic copper concentrations (scale bar = 50 µm). All fluorescence images were acquired with a 561 nm laser and have identical exposure times and normalization. (b) Brightfield images of the evolution of protrusions examples in each condition. The time between each image is 2 min, with a total interval of 6 min. The images are an extract from time‐lapses after 9 days of treatment with a total duration of 12 h. Arrows point to example protrusions and their evolution depending on treatment. Scale bar is 25 µm. ImageJ analyzed protrusions of six different spheroids per treatment to obtain statistical analysis of (c) length and (d) persistence through time. Data shown as its distribution with median and the interquartile range (IQR) (*n* = 6 spheroids per condition); ^*^ *p*‐val < 0.033; ^**^ *p*‐val < 0.002; ^***^ *p*‐val < 0.001.

We next investigated how copper exposure influences spheroid architecture and invasive potential. Specifically, we analyzed the actin cytoskeleton organization and the dynamics of protrusion formation. Using cytoskeletal staining, 3D reconstructions, and live imaging, we identified significant structural disruptions and impaired protrusive activity in copper‐treated spheroids, further revealing the impact of ionic copper at the sub‐cytotoxic level. We focused on the intermediate copper concentrations (0.025 and 0.01 mg mL^−1^), selected to study sub‐cytotoxic effects while maintaining biological relevance. The highest and lowest concentrations were excluded from this analysis due to their strong cytotoxicity or minimal effect, respectively. Representative 2D images of the cytoskeletal architecture of spheroids stained with phalloidin are shown in Figure S5a. Control spheroids showed robust growth with numerous matrix‐invading protrusions, often forming interconnections between neighboring spheroids. In contrast, copper‐treated spheroids showed disrupted actin networks, including the appearance of internal voids and peripheral actin accumulation, suggesting the formation of a dense encapsulating shell. Interestingly, a marked reduction in both the number and the extension of protrusions was appreciated. This effect was particularly pronounced in the 3D reconstructions shown in Figure 3a. While the nuclei appeared generally unaffected by copper treatment, slight morphological alterations were observed, as detailed in Figure S5a.

Time‐lapse imaging confirmed the inhibitory effect of copper on spheroid invasiveness (Figure 3b and Supporting Information Videos). In control conditions (Video S1), protrusions were highly dynamic and persistent over time. However, as copper concentration increased, protrusive activity progressively diminished, becoming nearly absent at the higher dose studied (Video S2 and S3). Although no significant differences were observed in the total number of protrusions formed (Figure S6a), as expected from the inherent behavior of spheroids to continuously attempt extension [34], their structure and function were severely compromised by copper exposure. Quantitative analysis of the time‐lapse sequences revealed a significant decrease in protrusion length following copper treatment (Figure 3c), with both concentrations showing a clear reduction compared to the control. Furthermore, protrusion persistence was also strongly affected (Figure 3d), while protrusions in control spheroids remained stable throughout the observation period, those in copper‐treated spheroids were short‐lived, rapidly retracting, likely due to an unfavorable microenvironment. Notably, the 0.025 mg mL^−1^ treatment condition showed highly significant differences compared to the control, while the 0.01 mg mL^−1^ condition showed a milder but still significant effect.

### 3.4 Invasive Behavior in Pre‐Formed Spheroids Exposed to Copper

**FIGURE 4:**
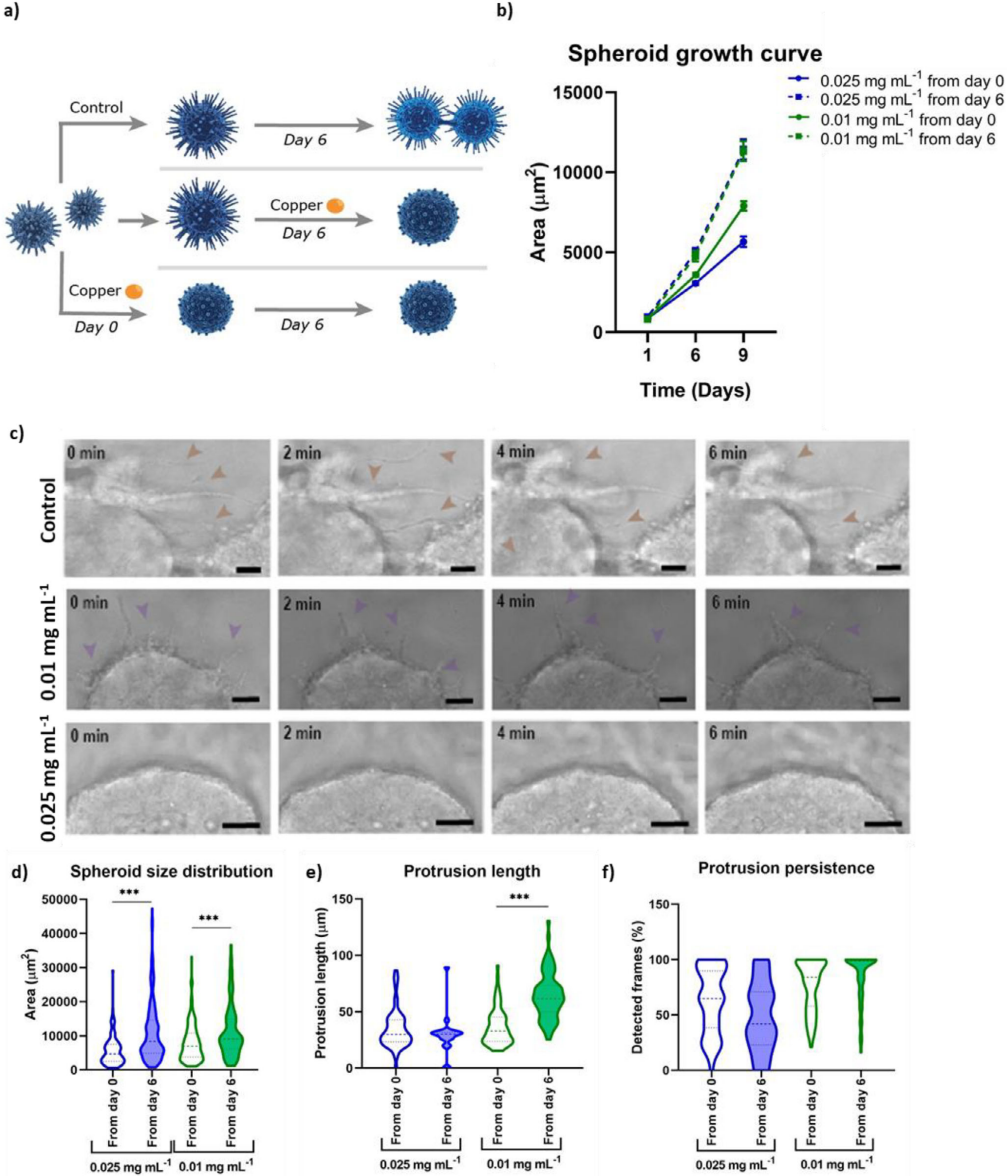
Influence of ionic copper treatment in preformed spheroids: (a) Schematic illustration of protrusion formation under different treatment conditions, (b) Growth curve at endpoint of spheroids over time under different treatment conditions. (c) Brightfield images illustrating the evolution of protrusions examples in each condition. The time between each image is 2 min, with a total interval of 6 min. The images are an extract from time‐lapses after 9 days of treatment with a total duration of 12 h. Arrows point to example protrusions and their evolution depending on treatment. Scale bar is 25 µm. ImageJ analyzed protrusions of six different spheroids per treatment to obtain statistical analysis of (d) length and (e) persistence through time; (f) population size distribution of spheroids at endpoint. Data shown as its distribution with median and the interquartile range (IQR) (*n* = 6 spheroids per condition); ^*^ *p*‐val < 0.033; ^**^ *p*‐val < 0.002; ^***^ *p*‐val < 0.001.

To further investigate copper's impact on established tumor structures, we conducted a complementary assay where copper was administered on day 6, after the spheroid formation (Figure 4a). While all copper concentrations were tested, only the highest copper concentration (0.05 mg mL^−1^) induced a statistically significant reduction in spheroid size by day 9 compared to controls (Figure S7), despite overall size reduction across all conditions. Figure 4b compares the growth curves of spheroids treated from day 0 versus day 6 with the intermediate copper concentrations (0.025 and 0.01 mg mL^−1^). Delaying copper exposure until day 6 led to larger spheroid sizes compared to early treatment. This trend was further confirmed by endpoint size distributions (day 9, Figure 4d), where day 6–treated spheroids showed greater variability and significantly larger sizes than their day 0 counterparts did. Given the distinct response observed depending on the timing of treatment, stress pathways were further investigated (Figure S4). Analysis of HIF‐1α expression revealed a dose‐ and time‐dependent response, with the highest expression detected in spheroids treated with 0.025 mg mL^−^^1^ copper at day 6, whereas lower copper concentrations at late time points resulted in reduced HIF‐1α levels. In addition, metabolic activity was slightly decreased compared to spheroids treated from day 0.

Structurally, 2D cytoskeletal imaging (Figure S5b) showed that spheroids treated at day 6 remained relatively compact and, notably for the 0.025 mg mL^−1^ treatment, often formed aggregates of multiple spheroids. Moreover, an actin capsule was visible around spheroids, suggesting cytoskeletal adaptation to the late copper exposure. However, 3D reconstructions (Figure S8) revealed a severely reduced protrusive activity: spheroids treated with 0.025 mg mL^−1^ at day 6 exhibited almost no protrusions, while those treated with 0.01 mg mL^−1^ showed a slightly higher number. At 0.05 mg mL^−1^, spheroids treated at day 6 were completely spherical, lacking protrusions and exhibiting a disrupted actin capsule. This alteration may be indicative of the presence of apoptotic vesicles surrounding the spheroids (Figure S5b). Live imaging supported these findings (Figure 4b): time‐lapse sequences showed persistent protrusions in controls, while those exposed to copper, particularly at higher doses, progressively lost protrusive activity. Although the number of protrusions was reduced (as shown in Figure S6b), no statistically significant differences were observed. When analyzing protrusion length (Figure 4e), we found that for the 0.025 mg mL^−1^ treatment, there was no significant difference between treatments initiated at day 0 and at day 6, indicating that, although spheroid growth was less compromised, protrusion dynamics were still affected. In contrast, in spheroids treated with 0.01 mg mL^−1^, protrusion length significantly increased when treatment was applied on day 6 compared to day 0, suggesting a partial preservation of invasive properties. Persistence analysis (Figure 4f) revealed no improvement in protrusion stability with delayed treatment, indicating that copper compromised persistence regardless of timing. To further explore spheroid protrusions, we assessed vimentin expression (Figure S11). In control spheroids, vimentin was strongly expressed throughout the cells, especially at the periphery and along protrusions, whereas all treatment conditions showed reduced expression, with only occasional staining at the spheroid edges and in sporadic protrusions. These results suggest that while delayed copper exposure allows for significant spheroid growth, it still impairs critical invasive behavior in glioblastoma spheroids. In addition, a qualitative assessment was conducted to examine the impact of copper treatment on the collagen hydrogel and its potential remodeling by tumor cells (Figure S10). Overall, no major alterations in collagen organization were observed across conditions; however, in specific treatments, a preferential orientation of collagen fibers became apparent.

### 3.5 Evaluation of Metastatic Potential via Cadherin Markers in Copper‐Treated Spheroids

**FIGURE 5:**
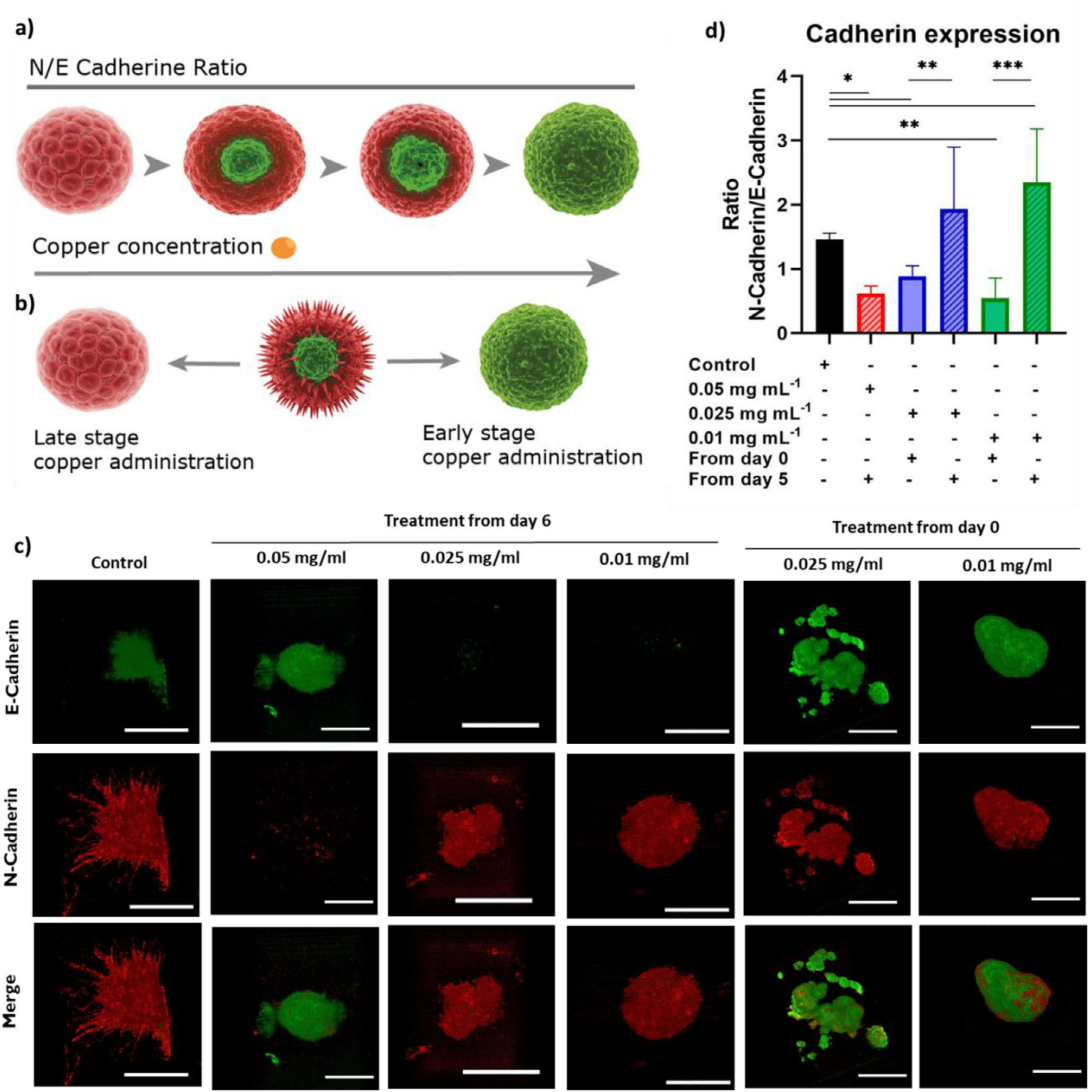
Malignancy reduction of 3D‐spheroids treated with ionic copper. (a) Illustration of N/E cadherin ratio evolution in spheroids treated with increasing concentrations of copper. (b) Illustration of N/E cadherin ratio in spheroids treated with copper at different stages. (c) 3D reconstruction of E‐ (green) and N‐Cadherin (red) expression and distribution over the spheroid (scale bar = 20 µm) when treated with ionic copper from day 6 or from day 0. All fluorescence images were acquired with 488 nm (green) and 640 nm (red) lasers and have identical exposure times and normalization. (d) Fluorescence intensity of the orthogonal projection was analyzed with Matlab to obtain the ratio of expression under the different treatments. Data shown as the mean ± SD (*n* = 3 spheroids at leats per condition); ^*^ *p*‐val < 0.033; ^**^ *p*‐val < 0.002; ^***^ *p*‐val < 0.001.

To assess how copper exposure influences glioblastoma aggressiveness, we evaluated the Epithelial‐to‐Mesenchymal Transition (EMT) status by analyzing the expression of E‐Cadherin and N‐Cadherin, two canonical markers of epithelial and mesenchymal phenotypes, respectively [35] (Figure 5a). Notably, in the untreated control group, spheroids exhibited a compact inner region with moderate E‐Cadherin (green) expression surrounded by a dominant N‐Cadherin (red) outer layer (Figure 5b). This spatial distribution is associated with an enhanced invasiveness and a more aggressive phenotype [36]. When spheroids were treated from day 0 with ionic copper at concentrations of 0.025 mg mL^−1^ or 0.01 mg mL^−1^, a marked increase in E‐Cadherin expression was observed, accompanied by a strong reduction in N‐Cadherin signal. This shift toward an epithelial phenotype was statistically significant compared to the control, indicating a suppression of EMT‐related invasive traits. Conversely, when intermediate copper concentrations (0.01 and 0.025 mg mL^−^^1^) were administered at day 6, after the spheroids were already formed, the pattern was reversed. In these conditions, E‐Cadherin expression was nearly absent, while N‐Cadherin dominated throughout the spheroid structure. This change was significantly different from both the untreated control and the corresponding day 0 treatments, suggesting an enhanced mesenchymal state (Figure 5c).

Interestingly, this mesenchymal shift did not occur when pre‐formed spheroids were treated on day 6 with the highest concentration of 0.05 mg mL^−1^ copper. Instead, these spheroids showed a strong epithelial phenotype characterized by high E‐Cadherin and low N‐Cadherin expression levels (Figure 5b). These findings suggest a dose‐dependent effect: higher copper concentrations may override mesenchymal programming and induce cytotoxicity in established spheroids, while lower doses may inadvertently enhance invasive potential.

## 4 Discussion

In this study, we demonstrate that extracellular collagen binds and retains copper ions, significantly affecting and influencing tumor spheroid behavior and structure. These findings offer new insights into the role of copper beyond its well‐established cytotoxic properties [11]. The uptake of Cu cations by cells is strictly regulated (i.e., STEAP or CTR1‐ mediated) [6, 13] and therefore recent studies [14, 24, 37] have used Cu‐containing nanoparticles that enter cells through endocytosis or pinocytosis pathways to produce a massive disruption in intracellular Cu levels, leading to cell death. In contrast, in this work, we show that a more nuanced regulation of Cu availability can produce interesting effects on the evolution of cancer cell spheroids. Specifically, copper‐ECM interactions were shown to modulate spheroid growth and invasiveness, thereby suggesting novel mechanisms by which copper can regulate tumor progression. One key observation is that high copper concentrations (observed at 0.05 mg mL^−1^) limit spheroid growth, being consistent with its cytotoxic effect reported elsewhere [14, 17].

However, the most interesting findings refer to the experiments with intermediate copper levels (0.025 and 0.01 mg mL^−1^) that, while displaying a lower cytotoxicity, still resulted in significantly suppressed invasiveness by impairing protrusion formation and cytoskeletal organization. Protrusions are essential for tumor invasion and metastasis, functioning as mechanosensory and signaling structures that facilitate ECM penetration and dissemination [38]. This inhibitory effect was most pronounced at 0.025 mg mL^−1^, where cytoskeletal disruption was accompanied by a marked reduction in protrusion persistence. This reduction in protrusion length and duration points to a functional shift in spheroid behavior, potentially induced by copper‐mediated stress responses. Protrusion persistence is essential for microenvironmental navigation and remodeling [34, 39]. Its disruption by copper may therefore represent a mechanism to suppress cell motility and metastatic potential. This observed reduction in protrusive activity upon copper treatment may be associated with altered focal adhesion turnover, which can limit cell edge dynamics and migration [40]. Additionally, copper could modulate Rho GTPase–mediated signaling pathways, affecting actin cytoskeleton remodeling and the formation of cellular protrusions, as suggested by the changes in vimentin distribution [34]. Altogether, these results reinforce the notion that controlling the invasive machinery of tumor cells, even without inducing cell death, may offer a valuable therapeutic alternative. At non‐cytotoxic levels, copper may emerge as a modulator of invasion via effects on cytoskeletal dynamics and protrusive structures.

Notably, the timing of copper exposure emerged as a critical factor influencing its effects. Copper also modulated EMT status, promoting an epithelial phenotype at high (more than 0.025 mg mL^−1^) copper concentrations or early‐stage doses, while potentially enhancing invasiveness at intermediate concentrations administered during later tumor stages (day 6). These results strongly align with previous reports in the literature, where copper ions can function as either a pro‐metastatic trace element or a therapeutic agent, depending on dose and timing [11]. Thus, previous studies have reported a pro‐tumorigenic role of copper, linking its bioavailability to enhanced EMT, AKT signaling, and increased invasive capacity in cancer cells. In this context, copper has been shown to modulate metalloproteinase activity and the shedding of adhesion molecules, thereby promoting a mesenchymal phenotype and cell dissemination [41, 42]. However, early copper treatment or higher concentrations impair metalloproteinase activity, ultimately restricting invasive behavior [43]. This suggests that while copper can promote mesenchymal programming under permissive conditions, excessive or premature exposure overrides these signaling effects by mechanically constraining the cells.

While the precise molecular mechanisms underlying copper‐induced phenotypic changes were not directly investigated in this study, our findings provide several indications pointing to potential pathways involved. The observed reduction in metabolic activity suggests that copper exposure may primarily affect cellular metabolism rather than triggering a classical oxidative stress–driven cell death program. In this context, recent reports on cuproptosis propose a mitochondria‐centered mechanism in which copper disrupts metabolic enzymes of the tricarboxylic acid cycle, leading to impaired cellular function [11, 44]. While further validation would be required, this framework is consistent with the reduced metabolic activity detected in copper‐treated spheroids. Together, these observations support a multifactorial mechanism in which metabolic perturbation, cytoskeletal regulation, and EMT modulation collectively contribute to the copper‐induced phenotypic changes, highlighting key biological processes that can be further explored at the molecular level.

In summary, copper holds therapeutic potential in modulating the TME, but precise control of dose and timing becomes essential to prevent unintended pro‐tumorigenic effects. While the collagen matrix displays a strong affinity for Cu, sequestering a large proportion of the Cu cations available in solution, it is still able to regulate spheroid behavior, likely by establishing a local equilibrium around the cells that modifies the local availability and supply of Cu. Our 3D model offers a useful platform to identify context‐specific windows of therapeutic efficacy and to better understand copper's influence on tumor behavior. The inherent affinity of copper for collagen and its capacity to influence spheroid dynamics introduce novel alternatives for targeting the ECM as a complementary cancer therapy.

## Conflicts of Interest

The authors declare no conflict of interest.

## Supporting information




**Supporting File**: adhm71131‐sup‐0001‐SuppMat.docx.

**Supporting Video 1**: adhm71131‐sup‐0002‐VideoS1.avi.

**Supporting Video 2**: adhm71131‐sup‐0003‐VideoS2.avi.

**Supporting Video 3**: adhm71131‐sup‐0004‐VideoS3.avi.

**Supporting Video 4**: adhm71131‐sup‐0005‐VideoS4.avi.

**Supporting Video 5**: adhm71131‐sup‐0006‐VideoS5.avi.

## Data Availability

The data that support the findings of this study are available in the supplementary material of this article.
