# Supplementary material for: Copper–Collagen Interactions Regulate the Mechanical and Invasive Properties of Tumor Spheroids
Source: Adv Healthc Mater. 2026 Apr 9;15(22):e05120. doi: 10.1002/adhm.202505120 (PMC13279867; doi:10.1002/adhm.202505120)
Supplement: Supplementary file 1 — Supporting File: adhm71131‐sup‐0001‐SuppMat.docx. [file ADHM-15-0-s006.docx]

Supplementary Information

**Copper–collagen interactions regulate mechanical and invasive properties of three-dimensional spheroids**

Paula Guerrero-López⁑, Jose I. Garcia-Peiro⁑*, Felipe Hornos, Jose L. Hueso, Jesus Santamaria and J. Manuel Garcia-Aznar*

J.I. Garcia Peiro, F. Hornos, J.L. Hueso, J. Santamaria

Instituto de Nanociencia y Materiales de Aragon (INMA); CSIC-Universidad de Zaragoza, Campus Rio Ebro, Edificio I+D, C/ Poeta Mariano Esquillor, s/n, 50018, Zaragoza, Spain.

J.I. Garcia Peiro, J.L. Hueso, J. Santamaria

Department of Chemical and Environmental Engineering; University of Zaragoza, Spain; Campus Rio Ebro, C/María de Luna, 3, 50018 Zaragoza, Spain.

J.I. Garcia Peiro, J.L. Hueso, J. Santamaria

Networking Research Center in Biomaterials, Bioengineering and Nanomedicine (CIBER-BBN), Instituto de Salud Carlos III; 28029 Madrid, Spain.

J.I. Garcia Peiro, J.L. Hueso, J.M. Garcia-Aznar, J. Santamaria

Instituto de Investigación Sanitaria (IIS) de Aragón, Avenida San Juan Bosco, 13, 50009 Zaragoza, Spain.

P. Guerrero-Lopez, J.M. Garcia-Aznar

Multiscale in Mechanical and Biological Engineering (M2BE); Aragon Institute of Engineering Research (I3A), University of Zaragoza, Mariano Esquillor s/n, 50018, Zaragoza, Spain.

Jose L. Hueso

Escuela Politécnica Superior, Universidad de Zaragoza, Crta. de Cuarte s/n, 22071, Huesca, Spain.

⁑ Equal contribution

*E-mail: [jmgaraz@unizar.es](mailto:jmgaraz@unizar.es) / [joseignacio.garcia@unizar.es](mailto:joseignacio.garcia@unizar.es)

Keywords: Copper, collagen, Microchips, 3D cultures, Cancer


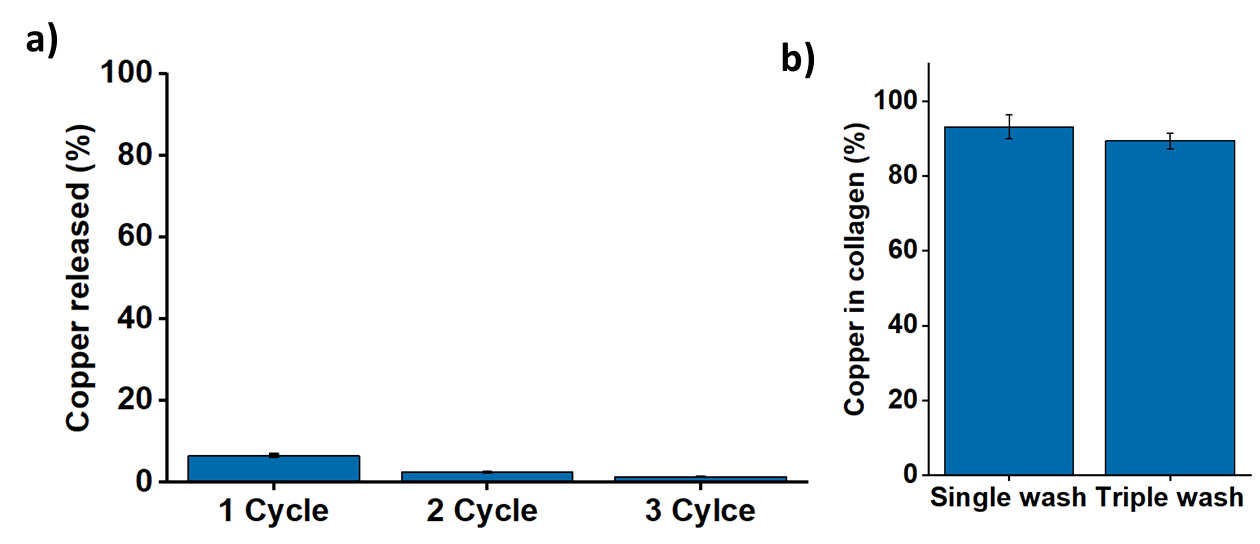


**Supplementary Figure 1. Retention and release of copper by collagen after sequential rinsing*.*** (a) Percentage of copper released from collagen after one, two, or three rinse cycles with water (8 hours release time). (b) Copper retained in collagen after one or three rinses, expressed as percentage of the initial copper content.

*
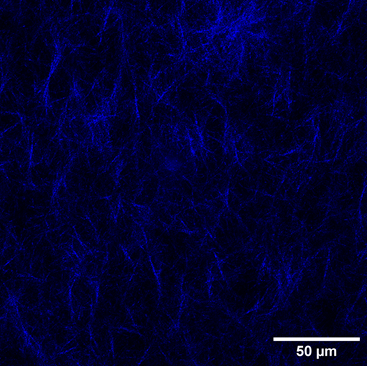
*

**Supplementary Figure 2. Image acquisition of collagen-based hydrogels using** reflection microscopy.

*
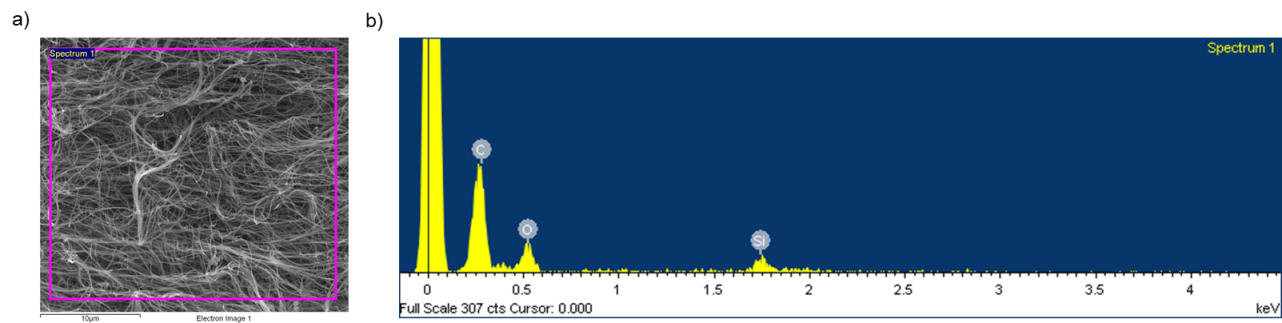
*

**Supplementary Figure 3. Cryo-**SEM **image of collagen-based hydrogels** and the corresponding SEM-EDX elemental analysis.

**
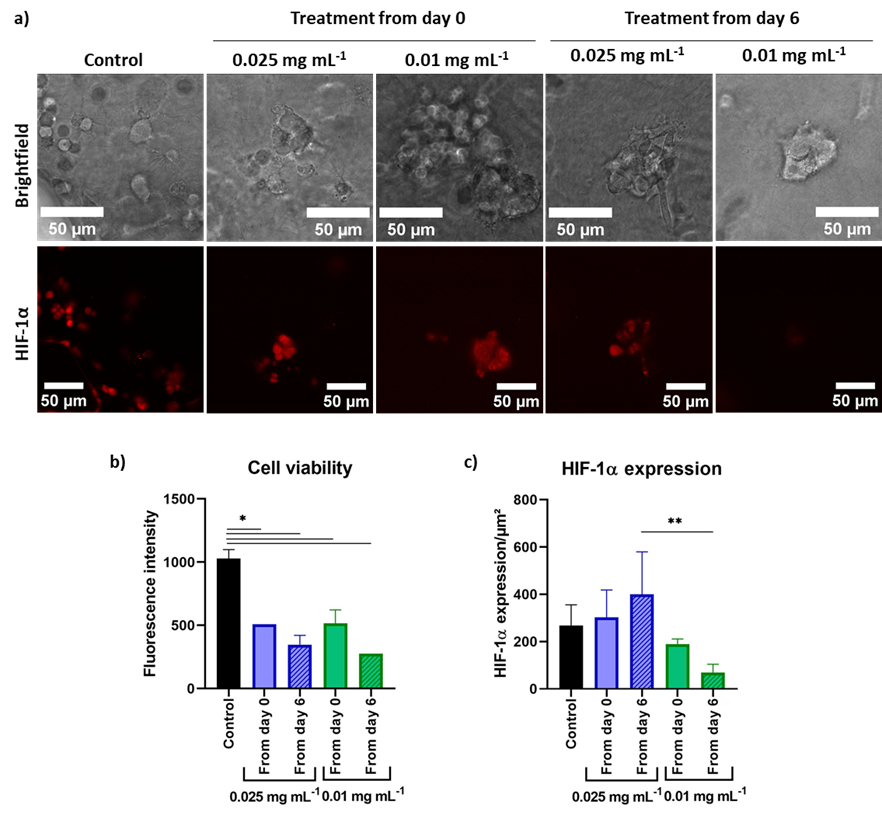
**

**Supplementary Figure 4. Assessment of cellular stress and metabolic activity in copper-treated spheroids.** a) Representative fluorescence images of HIF-1α expression in spheroids under the indicated copper treatments. All fluorescence images were acquired with an excitation of 488 nm end an emission of 610 nm and have identical exposure times and normalization. Scale bar: 50 μm. b) Cell viability/metabolic activity quantified using the Alamar Blue assay (n = 2). c) Quantification of HIF-1α expression, normalized to spheroid area (n = at least 4 spheroids per condition). Data are presented as mean ± SD. Statistical significance is indicated as *p-val < 0.033; **p-val < 0.002; ***p-val < 0.001.


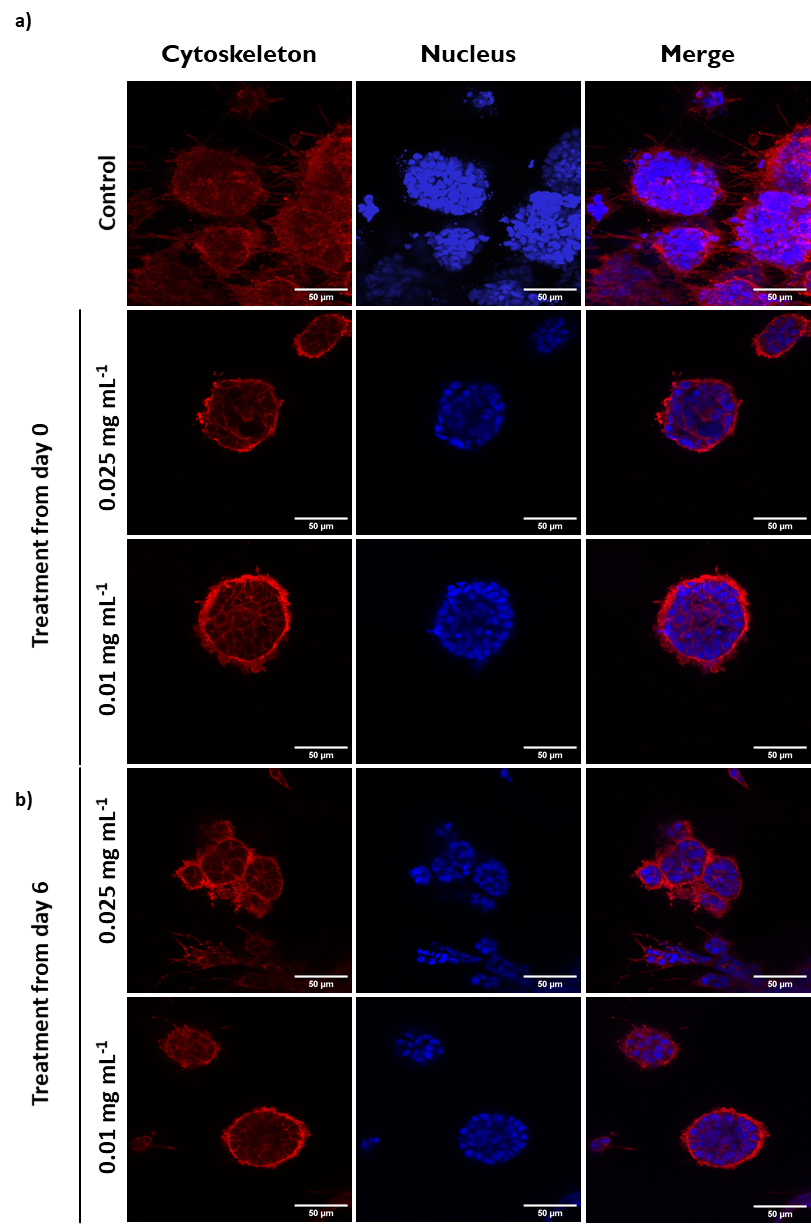


**Supplementary Figure 5.** 2D confocal images of GBM spheroids treated with different ionic copper concentrations: Nuclei was stained with Dapi (blue) and the cytoskeleton was stained with phalloidin (red); Scale bar 50 µm. All fluorescence images were acquired with 561nm laser and have identical exposure times and normalization.


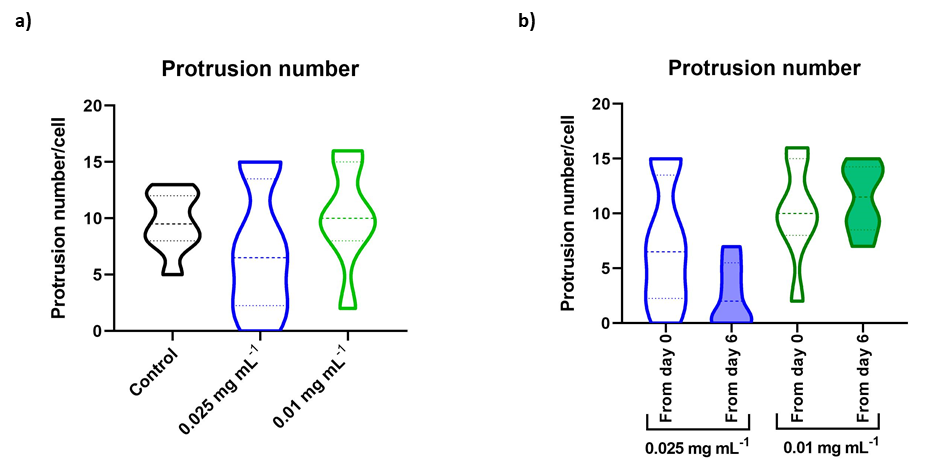


**Supplementary Figure 6:** Protrusion number produced under different ionic copper treatments. a) Protrusion number per spheroid when treated with 0.025 or 0.01 mg mL^-1^ ionic copper from the beginning. b) Comparison of the protrusion number per spheroid when treated with 0.025 or 0.01 mg mL^-1^ ionic copper at day 0 or 6. Data shown as its distribution with median and the interquartile range (IQR) (n = 6 spheroids per condition); **p*-val < 0.033; ***p*-val < 0.002; ****p*-val < 0.001.


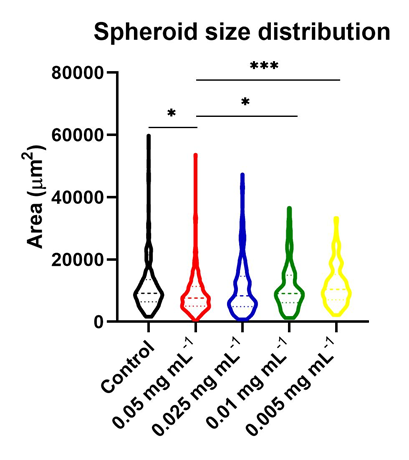


**Supplementary Figure 7.** Influence of ionic copper treatment in preformed spheroids: Population size distribution at endpoint. Data shown as its distribution with median and the interquartile range (IQR) (n = 3 technical replicates, and n=2 experimental replicates); **p*-val < 0.033; ***p*-val < 0.002; ****p*-val < 0.001.


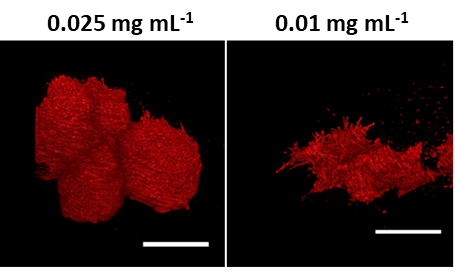


**Supplementary Figure 8:** 3D tumor reconstruction of confocal images of phalloidin stained GBM spheroids treated with 0.025 mg mL^-1^ or 0.01 mg mL^-1^ ionic copper at day 6 (scale bar = 50 µm).


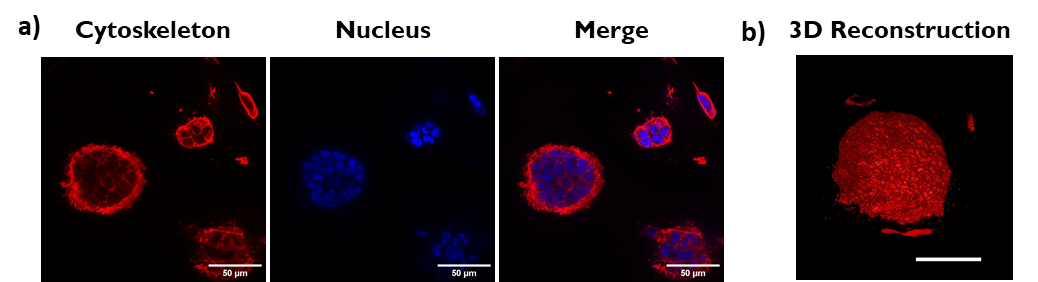


**Supplementary Figure 9.** Structure study of GBM spheroids treated with 0.05 mg mL^-1^ ionic copper from day 6: a) 2D confocal images of nuclei, stained with Dapi (blue), and the cytoskeleton, stained with phalloidin (red); Scale bar is 50 µm. b) 3D reconstruction of spheroid cytoskeleton. Scale bar is 50 µm.


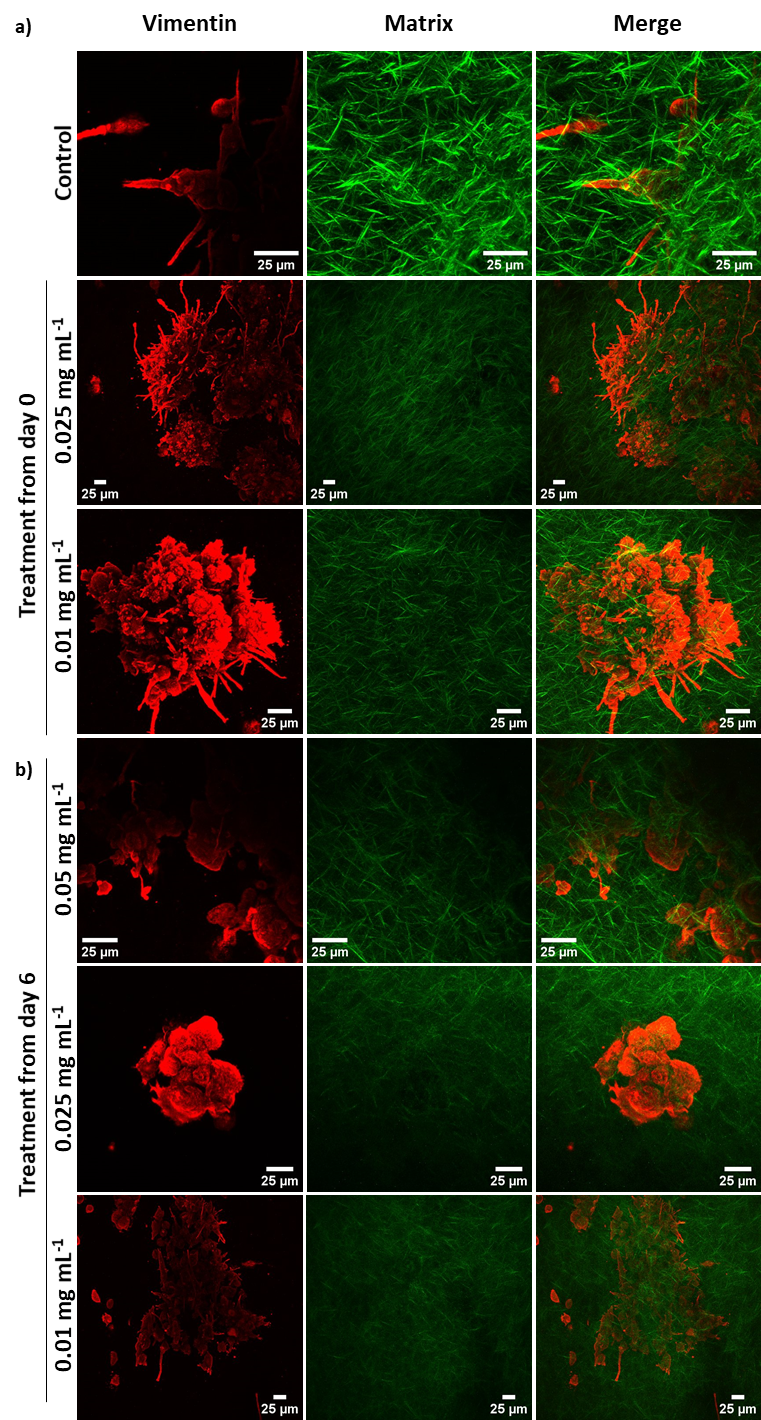


**Supplementary Figure 10. Collagen matrix organization under different copper treatment conditions.** Multiphoton microscopy images showing the collagen matrix surrounding tumor spheroids under control conditions and after copper treatment at 0.05, 0.025 and 0.01 mg mL-1 at day 0 (a) and 6 (b). Collagen was visualized by second-harmonic generation (SHG) multiphoton microscopy, while spheroids were stained for vimentin, acquired with 488nm laser and identical exposure times and normalization. Scale bar: 25 μm.


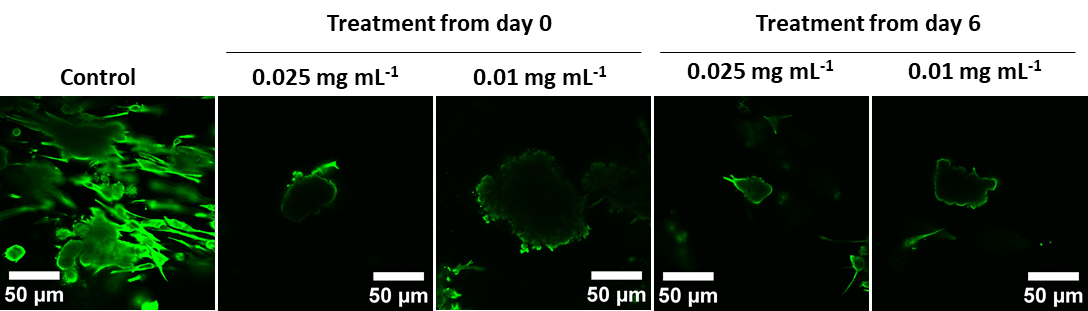


**Supplementary Figure 11. Vimentin expression.** Representative fluorescence images showing vimentin expression in spheroids under the indicated copper treatments. All fluorescence images were acquired with 488nm laser and have identical exposure times and normalization. Scale bar: 50 μm.
